# Extreme sensitivity of the electric-field-induced band gap to the electronic topological transition in sliding bilayer graphene

**DOI:** 10.1038/srep17490

**Published:** 2015-12-04

**Authors:** Kyu Won Lee, Cheol Eui Lee

**Affiliations:** 1 Department of Physics, Korea University, Seoul 136-713, Korea

## Abstract

We have investigated the effect of electronic topological transition on the electric field-induced band gap in sliding bilayer graphene by using the density functional theory calculations. The electric field-induced band gap was found to be extremely sensitive to the electronic topological transition. At the electronic topological transition induced by layer sliding, four Dirac cones in the Bernal-stacked bilayer graphene reduces to two Dirac cones with equal or unequal Dirac energies depending on the sliding direction. While the critical electric field required for the band gap opening increases with increasing lateral shift for the two Dirac cones with unequal Dirac energies, the critical field is essentially zero with or without a lateral shift for the two Dirac cones with equal Dirac energies. The critical field is determined by the Dirac energy difference and the electronic screening effect. The electronic screening effect was also found to be enhanced with increasing lateral shift, apparently indicating that the massless helical and massive chiral fermions are responsible for the perfect and imperfect electronic screening, respectively.

Bilayer graphene (BLG) has been recently predicted to undergo an electronic topological transition under a tiny lateral interlayer shift, which may alter the physical properties of BLG significantly^1^. At the electronic topological transition induced by layer sliding, four Dirac cones in the AB-stacked BLG reduces to two Dirac cones with equal or unequal Dirac energies depending on the sliding direction^1^. A tiny lateral shift, little modifying the binding energy and the interalyer distance of the AB-stacked BLG, is expected to occur easily in real samples^1^. Stacking faults by layer sliding were found in recent experiments on BLG grown by chemical vapor deposition at the domain walls between two ideally stacked graphene systems^2^,^3^,^4^, and sliding of graphene flakes may be initiated by the scanning tunneling microscopy tip^5^. The optimal configuration of the BLG nanoribbons has recently been reported to have a lateral shift between the layers^6^.

On the other hand, the band gap in BLG can be controlled by an external electric field perpendicular to the layer^7^,^8^,^9^,^10^, providing a very useful way for graphene field effect devices. While an anomalous optical phonon splitting in sliding BLG may be utilized to detect tiny misalignment of graphene layers by using the spectroscopic tools^11^, the effect of layer sliding on the electric field-induced band gap in BLG, which is crucial for the characterization and application of the gated BLG, need to be clarified. Here, our density functional theory (DFT) calculations show that the electric field-induced band gap is extremely sensitive to the electronic topological transition induced by layer sliding in BLG. While the critical electric field required for the band gap opening increases with increasing lateral shift for the two Dirac cones with unequal Dirac energies, the critical field remains essentially zero even with a lateral shift for the two Dirac cones with equal Dirac energies.

The AB and BA stackings, the Bernal stackings, are equivalent to each other, with the carbon atoms of a layer residing on top of a carbon atom or a hollow center of the other layer. In the AA stacking, all the carbon atoms of a layer reside on top of the carbon atoms of the other layer and, in the AA′ stacking, all the carbon atoms of a layer reside on the hollow of the other layer^12^. The electronic structure of AB-stacked BLG has a quadratic energy dispersion around the *K* point of the first Brillouin zone, which changes to four Dirac cones with linear dispersions in the very near neighbor of the *K* point^13^. The electronic structures of AA- and AA′-stacked BLG have just two Dirac cones with unequal Dirac energies around the *K* point^12^. The Dirac energy and the Dirac point respectively indicate the energy and the wavevector when the upper and lower cones are in contact. In the AA stacking, two vertical Dirac cones with unequal Dirac energies have the same Dirac point and, in the AA′ stacking, two nonvertical Dirac cones with unequal Dirac energies have different Dirac points^12^.

A tiny lateral interlayer shift in the AB-stacked BLG has been predicted to induce an electronic topological transitions in the low energy electronic structure, conserving the quadratic energy dispersion at a relatively high energy^1^. As the lateral shift increases further along the armchair direction, which is the shortest path from the AB to the BA stacking, the AB stacking changes to the AA or AA′ stacking, where the quadratic dispersion disappears^12^. Since the BA stacking is equivalent to the AB stacking, the lateral shift can be taken to occur along the path from the AB to the AA stacking or to the AA′ stacking. In this work, our DFT calculations show that the electronic screening effect increases with increasing lateral shift and, finally, the electric field would be perfectly screened in the AA and AA′ stackings, possibly indicating that the massless helical and massive chiral fermions are responsible for the perfect and imperfect electronic screening, respectively. Also, the critical electric electric field required for the band gap opening appears to be determined by the Dirac energy difference and the electronic screening effect.

Figure 1 shows the atomic geometry and the energy map for the valence band near the *K* point of the Brillouin zone. Fig. 1a,b correspond to AB-stacked BLG. Considering the low energy electronic structure of AB-stacked BLG^1^,^13^, four Dirac cones are formed around the Fermi level at the Dirac points of 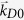, 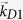, 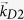, and 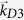 as shown in Fig. 1b. Figure 1c–f correspond to sliding BLG. In this work, the lower layer slides with respect to the fixed upper layer along the 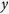-axis corresponding to the armchair direction as shown in Fig. 1c,e, which is described by a sliding vector 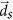 = 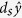. Figure 1c,d correspond to sliding BLG with *d*_*s*_ = +0.24 Å, and Fig. 1e,f correspond to sliding BLG with *d*_*s*_ = −0.24 Å. In Fig. 1c,e, the top and side views of sliding BLG are shown on the left and right, and the sliding direction of lower layer is indicated by arrows. AB-stacked BLG corresponds to *d*_*s*_ = 0. With the C-C bond length *a*_*c*_ = 1.42 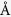, *d*_*s*_ = *a*_*c*_ or *d*_*s*_ = −2*a*_*c*_ give a BA stacking. On the other hand, *d*_*s*_ = 0.5*a*_*c*_ and *d*_*s*_ = −*a*_*c*_ give an AA′ and an AA stacking, respectively^12^. Thus, the layer sliding occurs along the path from the AB to the AA′ stacking when *d*_*s*_ > 0 or along the path from the AB to the AA stacking when *d*_*s*_ < 0. The layer sliding along the 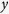-axis includes the two electronic topological transitions predicted in previous works^1^. When *d*_*s*_ + 0.012 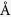, the three Dirac cones at 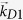, 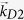, and 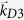 collapse into a single Dirac cones as shown in Fig. 1d. When *d*_*s*_ − 0.004 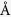, the two Dirac cones at 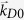 and 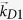 meet and disappear leaving only two Dirac cones at 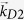 and 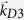 as shown in Fig. 1f. While the energy maxima in Fig. 1d corresponding to the Dirac energy show a difference between the Dirac cones at 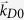 and 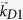, the maxima in Fig. 1f show no difference between the Dirac cones at 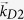 and 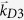
1. The electronic topological transition induced by layer sliding leads to two Dirac cones with equal or unequal Dirac energies depending on the sliding direction^1^,^12^, significantly changing the physical properties of BLG as will be discussed below.

Figure 2 shows the electric field-induced band gap obtained from the band structure and density of states (DOS) as a function of the electric field *F*_*z*_ perpendicular to the layer. In the AB-stacked BLG, the critical electric field *F*_*c*_ required for the band gap opening was about 10 mV/nm, as in previous works^14^. Figure 2a,b show the electric-field induced band gap when the layer sliding occurs along the path from the AB to the AA′ stacking (*d*_*s*_ > 0) and when the layer sliding occurs along the path from the AB to the AA stacking (*d*_*s*_ < 0), respectively. The most notable is that, while the critical field *F*_*c*_ increases with increasing lateral shift along the path from the AB to the AA′ stacking (*d*_*s*_ > 0, see Fig. 2a), the critical field *F*_*c*_ was less than 10 mV/nm and is believed to be essentially zero for any lateral shift along the path from the AB to the AA stacking (*d*_*s*_ < 0, see Fig. 2b). In other words, the critical field increases with increasing lateral shift for the two Dirac cones with unequal Dirac energies but is essentially zero with or without a lateral shift for the two Dirac cones with equal Dirac energies. The electric field-induced band gap is extremely sensitive to the electronic topological transition induced by layer sliding, which can be utilized to characterize the gated BLG and may be responsible for the contradicting results in recent experiments on the gated BLG^15^,^16^,^17^,^18^,^19^.

Figure 3 shows the band structures and DOS near the *K* point. In each panel, the band structure and DOS are shown on the left and right, respectively. The DOS of sliding BLG exhibits symmetric peaks around the Fermi level caused by the saddle points between the two Dirac cones, consistently with a previous work^12^. Figure 3a,b show the band structure at *F*_*z*_ = 1 and 3 V/nm, respectively, when *d*_*s*_ = +0.24 Å. The layer sliding along the path from the AB to the AA′ stacking (*d*_*s*_ > 0) produces two Dirac cones with unequal Dirac energies (see Fig. 1d). As shown in Fig. 3a, at *F*_*z*_ = 1 V/nm, an energy gap *ε* opens at the Dirac points but there is no band gap since *ε* is still smaller than the Dirac energy difference 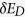 between the two Dirac cones. As shown in Fig. 3b, at *F*_*z*_ = 3 V/nm, *ε* larger than 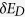 leads to a band gap *E*_*g*_. When *d*_*s*_ > 0, the electric field-induced energy gap *ε* must exceed the Dirac energy difference in order to open a band gap, leading to a finite critical field. On the other hand, the layer sliding along the path from the AB to the AA stacking produces two Dirac cones with equal Dirac energies (see Fig. 1f) and the electric field-induced energy gap 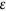, even if infinitesimally small, directly leads to a band gap when *d*_*s*_ < 0 as shown in Fig. 3c,d. The results will be further discussed below. Thus, the electronic phase produced by a layer sliding along the path from the AB to the AA stacking is believed to be very sensitive to a perturbation such as spin-orbit interaction or the substrate.

As can be seen in Fig. 2, the band gap is quite linear to the electric field *F*_*z*_. In the tight binding model for the AB-stacked BLG^13^, the electrostatic potential energy difference 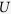 between the two layers gives rise to a band gap 
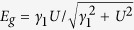
 with the nearest neighbor interlayer hopping parameter 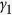. For a large *U* ≫ *γ*_1_, the band gap has a saturation value, 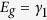. For a small *U* ≪ *γ*_1_, the band gap is equal to the potential energy difference, 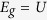. The potential energy difference 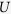 induced by the electric field *F*_*z*_ under an electronic screening effect can be described as 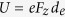. Here, 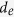 is the effective interlayer distance including the electronic screening effect and was about 1 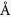 in the AB-stacked BLG^14^. 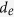 equal to the interlayer distance of BLG indicates that there is no screening effect, and 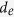 = 0 indicates that the electric field is perfectly screened and *E*_*g*_ = *U*  for any *F*_*z*_. Thus, the electric field-induced band gap can be described as 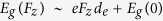, where the offset 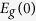 was added for the Dirac energy difference. The straight lines in Fig. 2 show the linear fits. When *d*_*s*_ < 0, the offset 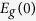 was set to zero. 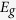 is not a simple linear function of 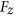 around *d*_*s*_  ~ − 0.71 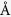 and when *d*_*s*_ ≳ 0.2 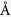 (see Fig. 2). When *d*_*s*_ ≳ 0.48 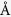, 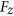 does not open a band gap because of the critical field high enough to close the energy gap between the 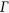 and 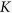 points even if an energy gap opens at the 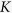 point.

Figure 4a,b show 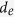 and 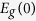, respectively, obtained by the linear fits in Fig. 2. The critical field *F*_*c*_ shown in Fig. 4c was obtained directly from Fig. 2. As shown in Fig. 4a, the effective interlayer distance *d*_*e*_ decreases with increasing *d*_*s*_ and appears to go to zero when *d*_*s*_ goes to −*a*_*c*_ or +0.5*a*_*c*_, each corresponding to the AA and AA′ stackings. The results indicate that the electronic screening effects increase as the sliding BLG approaches the AA or AA′ stacking. Indeed, we observed that the electric field *F*_*z*_ does not open an energy gap at the Dirac point in the AA′ stacking or in the AA stacking, indicating that the electric field is perfectly screened. For *d*_*s*_ = 0.48 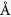, *F*_*z*_ does not open a band gap but still opens an energy gap increasing with the field at the Dirac points, indicating that the electric field is imperfectly screened. While, in a tight binding model for the AA-stacked BLG^20^, the electrostatic potential energy difference between the layers has already been reported not to open a band gap, our results indicate that the electric field is perfectly screened in the AA-stacked BLG and thus cannot induce a potential energy difference. The AA and AA′ stackings have just two Dirac cones with linear energy dispersion contrasting to the quadratic energy dispersion characteristic of the AB stacking. A previous work^21^ showed that when the AB-stacked BLG is decoupled by an increase of the interlayer distance and the electric field-induced charge redistribution, the electric field cannot open a band gap. Thus, the massless helical fermions with a linear energy dispersion would be responsible for the perfect screening while the massive chiral fermions with a quadratic dispersion would be responsible for the imperfect screening.

In Fig. 4b, the Dirac energy difference 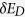 between the Dirac cones at 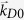 and 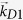, which is directly obtained from the energy map for the valence band such as shown in Fig. 1, was compared with 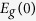 obtained by the linear fits in Fig. 2. In a previous tight binding model for the sliding BLG^1^, the Dirac energy difference between the Dirac cones at 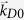 and 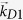 was described as 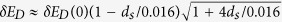 and there is no Dirac energy difference between the Dirac cones at 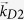 and 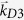. The solid line in Fig. 4b corresponds to the tight binding model description with 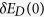 = 1 meV, which is consistent with our DFT results. 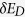 also agrees well with 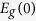, especially for small *d*_*s*_. Since the band gap is zero at the critical electric field *F*_*c*_, indicating that the critical field 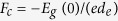 is determined by the Dirac energy difference and the electronic screening effects.

Experimental measurements of the band gap in BLG are still controversial^15^,^16^,^17^,^18^,^19^ and can be affected by a strain^22^, twist^23^, or a lateral shift. We did not find experimental measurements appropriate enough to be compared with our DFT results, however, while preparing this manuscript, a recent tight binding (TB) model study on a similar issue came to our attention^24^. Since the TB model includes only the first-nearest-interlayer hopping terms^24^ and the details of the electronic topological transition are determined by the higher-order interlayer hopping terms^1^, the electronic topological transition induced by a tiny lateral shift in the AB stacking and its effect on the electric field-induced band gap opening were not investigated in detail in the TB model. Instead, the TB model investigated a lateral shift in AA and AA′ stackings, each leading to two Dirac cones with equal and unequal Dirac energies, and the electric field-induced band gap opening was shown to be sensitive to the Dirac energy difference between the two Dirac cones, consistently with our DFT results. Despite the great success of DFT calculations for the electronic properties, the limits of DFT calculations to provide realistic estimation of the band gap have long been known and more realistic estimation of the band gap can be achieved by using the hybrid functionals and GW corrections. However, the essential characteristics of the electric field-induced band gap in sliding BLG, for which our DFT calculations are consistent with a TB model prediction, is believed to be unaffected.

In summary, we have investigated the effect of electronic topological transition on the electric field-induced band gap in BLG by using the DFT calculations. Under a lateral interalyer shift, AB-stacked BLG undergoes an electronic topological transition, where four Dirac cones around the Fermi level reduces to two Dirac cones with equal or unequal Dirac energies depending on the shift direction. While the critical electric field required for the band gap opening increases with increasing lateral shift for the two Dirac cones with unequal Dirac energies, the critical field is essentially zero even with a lateral shift for the two Dirac cones with equal Dirac energies. The critical field is determined by the Dirac energy difference and the electronic screening effect. The massless helical and the massive chiral fermions are believed to be responsible for perfect and imperfect electronic screening effect, respectively.

## Methods

A Bernal-stacked BLG was considered as a two-dimensional system with a lattice constant *a* = 2.46 Å and an interlayer distance 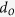 = 3.35 Å. For sliding BLG, the interlayer distance was taken from a previous work^1^. Our DFT calculations were carried out without considering the Van der Walls interaction. However, while the Van der Walls interaction between graphene layers plays an important role in determining the interlayer distance^1^, the electronic properties are little affected by it as would be our results^7^,^9^. The vacuum spacing between the BLGs was set to 20 Å. As usual, the 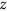 axis was taken along the direction perpendicular to the layer. A Quantum Espresso package, using plane waves as the basis set for wave functions, was employed for the DFT calculation^25^. Norm-conserving fully-relativistic pseudopotentials^26^ and a generalized gradient approximation (GGA) of Perdew-Burke-Ernzerhof (PBE)^27^ form for the exchange-correlation (XC) potential were used. Based on the stable charge density obtained from self consistent field (SCF) calculations with a kinetic energy cutoff of 250 Ry and 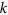-points of 48 × 48 × 1 mesh, the wavefunctions were obtained with 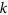-points of 24 × 24 × 1 mesh. Some calculations were done with 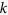-points of 96 × 96 × 1 mesh for SCF calculations and 48 × 48 × 1 mesh for wavefunctions to check for the reliability. A periodic sawtooth potential was used to simulate the external electric field. Using the wavefunctions obtained from the DFT calculations, relativistic maximally localized Wannier functions were constructed within the Wannier90 code^28^. Based on the Wannier interpolation, all the electronic properties such as the density of states were calculated.

## Additional Information

**How to cite this article**: Lee, K. W. and Lee, C. E. Extreme sensitivity of the electric-field-induced band gap to the electronic topological transition in sliding bilayer graphene. *Sci. Rep.* **5**, 17490; doi: 10.1038/srep17490 (2015).

## Figures and Tables

**Figure 1:**
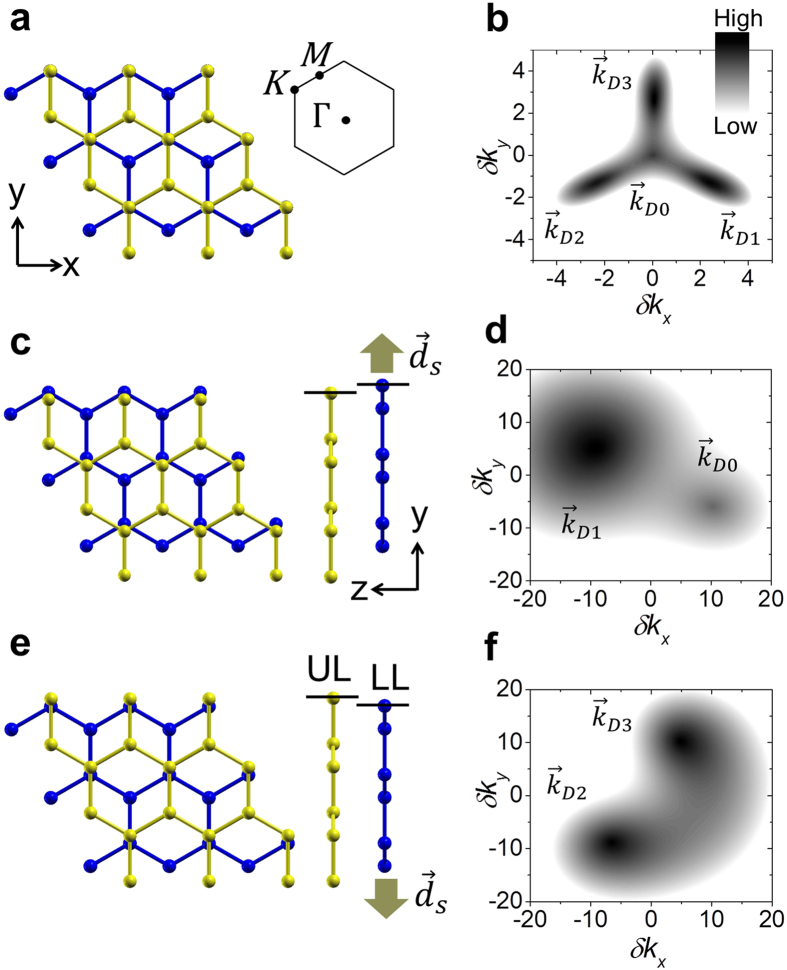
Atomic geometry and energy map for the valence band near the 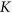 point. (**a**) Atomic geometry of AB-stacked BLG. Blue and yellow spheres correspond to the carbon atoms in the lower and upper layers, respectively. Inset shows the first Brillouin zone and the symmetric points of 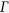, 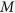 and 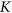. (**b**) Energy map for the valence band of AB-stacked BLG. 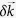 = 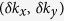 = 1000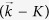 in units of 2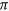/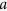 and high energy appears dark. The darkest spots (energy maxima) correspond to four Dirac cones at the Dirac points of 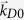, 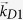, 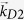 and 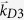. Panels (**c**,**d**) show the atomic geometry and the energy map for the valence band of sliding BLG with *d*_*s*_ = + 0.24 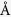, respectively. Panels (**e**,**f**) show the atomic geometry and the energy map for the valence band of sliding BLG with 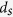 = −0.24 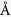, respectively. In panels (**c**,**e**), The top and side views are shown on the left and right, respectively, and the arrows indicate the sliding direction of the lower layer (LL) with respect to the fixed upper layer (UL).

**Figure 2:**
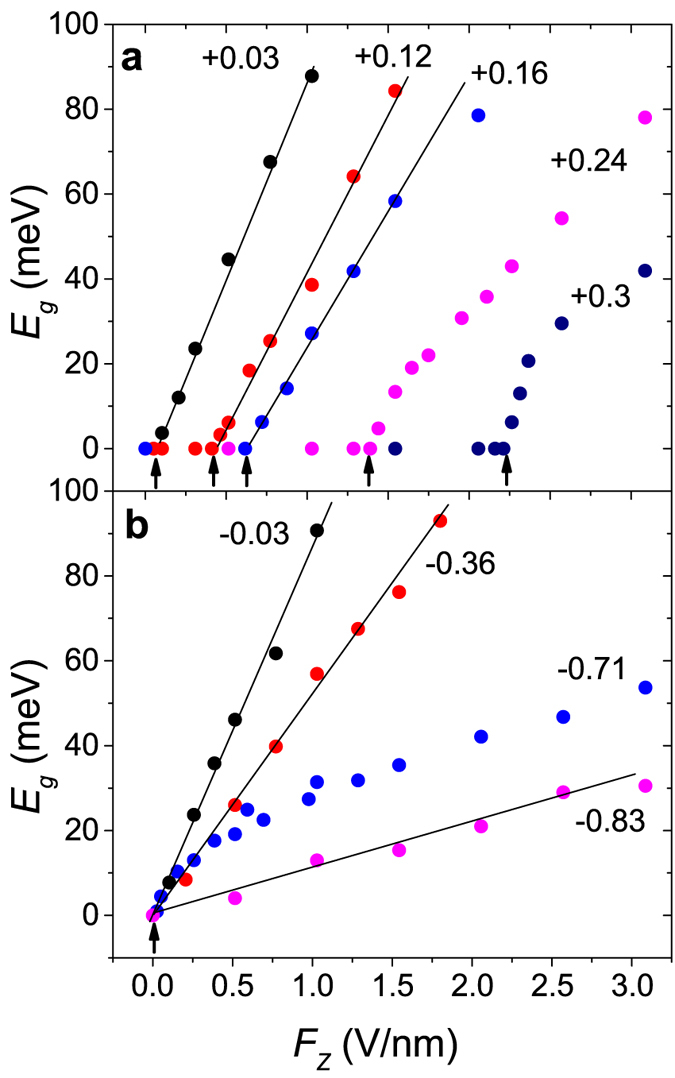
Electric field-induced band gap as a function of electric field 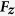. (**a**) When the layer slides along the path from the AB to the AA′ stacking (*d*_*s*_ > 0). (**b**) When the layer slides along the path from the AB to the AA stacking (*d*_*s*_ < 0). Numbers indicate *d*_*s*_ in units of 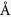 and arrows indicate the critical field *F*_*c*_ required for the band gap opening. The straight lines are the linear fits as described in the text.

**Figure 3:**
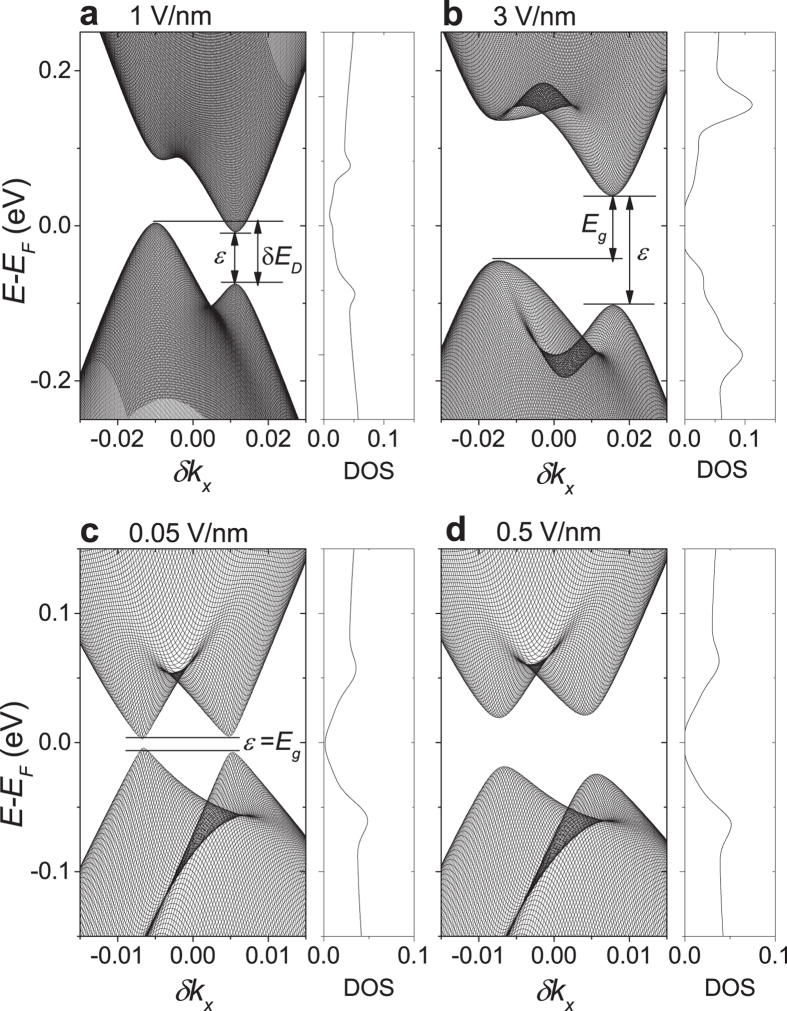
Band structure and density of states near the 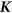 point. In each panel, the band structure and density of states (DOS) are shown on the left and right, respectively. The Fermi level 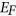 was set to zero. Panels (**a**,**b**) correspond to *F*_*z*_ = 1 and 3 V/nm, respectively, when *d*_*s*_ = +0.24 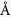. Panels (**c**,**d**) correspond to *F*_*z*_ = 0.05 and 0.5 V/nm, respectively, when *d*_*s*_ = −0.24 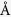. DOS was shown in arbitrary units and 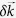 = 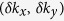 = 1000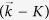 in units of 2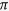/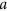. When *d*_*s*_ > 0, as shown in panels (**a**,**b**), the electric field-induced energy gap 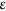 at the Dirac points must exceed the Dirac energy difference 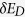 in order to open a band gap 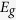. When *d*_*s*_ < 0, as shown in panels (**c**,**d**), 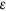 is equal to 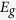.

**Figure 4:**
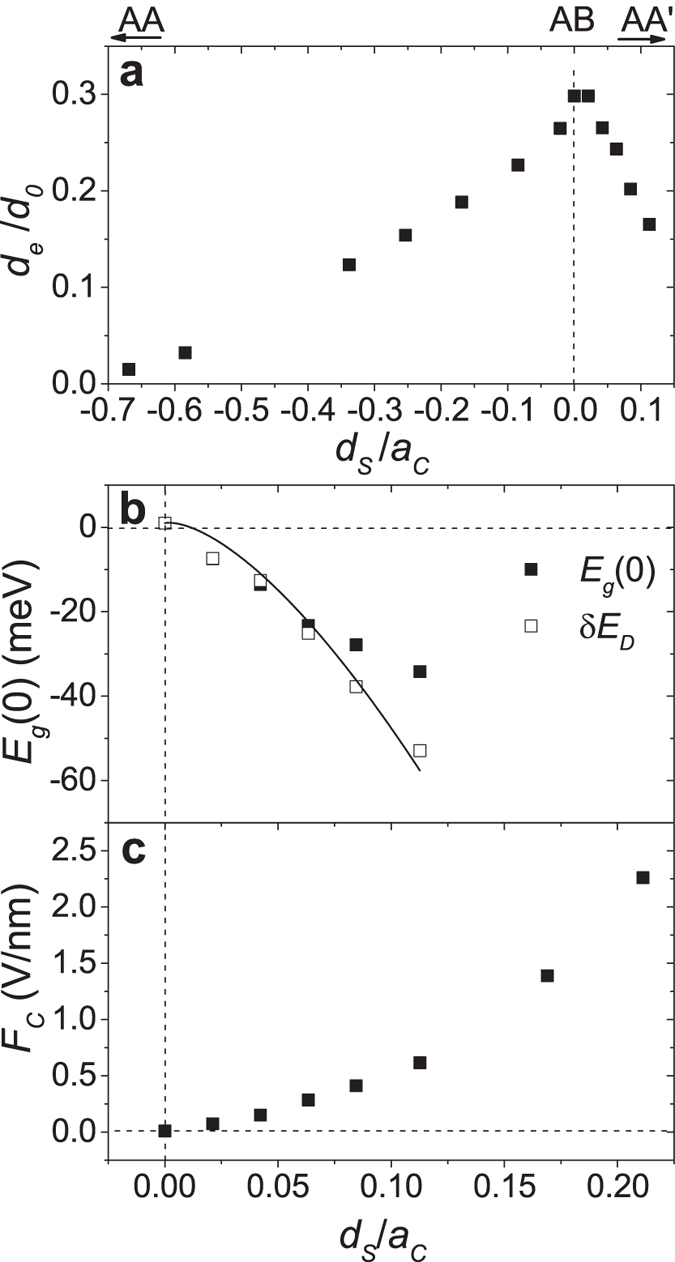
Parameters 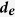, 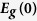 and 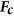 as a function of 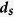/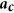. The C-C bond length 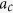 was set to 1.42 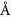. (**a**) Effective interlayer distance 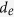 normalized to the interlayer distance 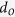 of the AB-stacked BLG. (**b**) The offset 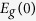 obtained from the linear fits in Fig. 2 is compared to the Dirac energy difference 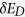 obtained from the energy map for the valence band such as shown in Fig. 1. The solid line in panel (**b**) corresponds to the tight binding model description for 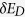 as explained in the text. (**c**) Critical electric field *F*_*c*_ obtained directly from Fig. 2.
